# Herbal Therapy for the Treatment of Acetaminophen-Associated Liver Injury: Recent Advances and Future Perspectives

**DOI:** 10.3389/fphar.2020.00313

**Published:** 2020-03-11

**Authors:** Ling Chang, Dongwei Xu, Jianjun Zhu, Guangbo Ge, Xiaoni Kong, Ying Zhou

**Affiliations:** ^1^Department of Gastroenterology, The Seventh People’s Hospital of Shanghai University of Traditional Chinese Medicine, Shanghai, China; ^2^Department of Liver Surgery, Renji Hospital, School of Medicine, Shanghai Jiao Tong University, Shanghai, China; ^3^Institute of Interdisciplinary Integrative Medicine Research, Shanghai University of Traditional Chinese Medicine, Shanghai, China; ^4^Central Laboratory, Department of Liver Diseases, Institute of Clinical Immunology, ShuGuang Hospital Affiliated to Shanghai University of Chinese Traditional Medicine, Shanghai, China

**Keywords:** herbal therapy, acetaminophen, liver injury, P450 enzyme, oxidative stress, gut microbiota

## Abstract

Acetaminophen (APAP) overdose is the leading cause of drug-induced liver injury worldwide, and mitochondrial oxidative stress is considered the major event responsible for APAP-associated liver injury (ALI). Despite the identification of N-acetyl cysteine, a reactive oxygen species scavenger that is regarded as an effective clinical treatment, therapeutic effectiveness remains limited due to rapid disease progression and diagnosis at a late phase, which leads to the need to explore various therapeutic approaches. Since the early 1990s, a number of natural products and herbs have been found to have hepatoprotective effects against APAP-induced hepatotoxicity in terms of acute liver failure prevention and therapeutic amelioration of ALI. In this review, we summarize the hepatoprotective effects and mechanisms of medicinal plants, including herbs and fruit extracts, along with future perspectives that may provide guidance to improve the current status of herbal therapy against ALI.

## Introduction

Acetaminophen (APAP) is a commonly used analgesic and antipyretic drug that is found in a number of combined prescriptions, including Tylenol with codeine and Hycotab (Aminoshariae and Khan, 2015). Although its effectiveness and safety were confirmed at recommended doses, the APAP overdose causes hepatotoxicity that leads to acute liver failure (ALF) (Bunchorntavakul and Reddy, 2018). According to the ALF Study Group in the United States, APAP-associated toxicity contributes to approximately 46% of all cases of ALF in adults, which far exceeds idiosyncratic drug-induced liver injury (DILI) by more than fourfolds (Ostapowicz et al., 2002; Lee, 2013).

Accumulating evidence has highlighted that APAP-induced oxidative stress and mitochondrial dysfunction are the fundamental factors in the pathogenesis of APAP-associated liver injury (ALI); thus, N-acetyl cysteine (NAC), a scavenger of reactive oxygen species (ROS), is considered a standard therapeutic option for APAP overdose (Jaeschke et al., 2012). However, due to the narrow therapeutic window, severe adverse effects, and rapid disease progression, the therapeutic efficacy of NAC is still limited (Du et al., 2016). For patients at the advanced stage, liver transplantation is the only way to improve survival outcomes (Craig et al., 2010). Therefore, new treatments that are superior to NAC in terms of therapeutic efficacy and safety are required in clinical practice.

Recently, herbal medicine was found to be a promising therapeutic approach for ALI. Several herbal components were reported to have the same therapeutic effect as NAC (Chen et al., 2009; Mukazayire et al., 2010; Tien et al., 2014). In a previous study, the effectiveness of the treatment was significantly noticed when it was administered after APAP, but not as a pretreatment. These underlying limitations regarding the applicability and effectiveness of herbal therapy have been modified and improved in recent years by direct application in an APAP overdose setting. In this review, we highlighted recent advances and new findings regarding the impact, effectiveness, and underlying mechanisms of herbal therapies in ALI.

### Underlying Mechanisms of APAP and ALI

The majority of APAP is conjugated with glucuronic acid and then excreted through the kidney when consumed at therapeutic doses. The remnants of the dose form reactive metabolites (N-acetyl-p-benzoquinone imine; NAPQI) by cytochrome P450 enzymes (McGill and Jaeschke, 2013). Although the reactiveness of NAPQI is high, it is barely harmful at the therapeutic dose because of its abundant glutathione storage and excretion through bile. In contrast, an overdose of APAP causes hepatotoxicity by saturating the sulfation pathway and generating NAPQI that leads to a robust reaction between NAPQI and hepatic glutathione stores, which subsequently depletes glutathione (Xie et al., 2015).

Currently, APAP protein adduct formation and release into the circulation is being utilized as a biomarker for APAP overdose (Davern et al., 2006). However, these molecules are also identified in the majority of subjects taking a therapeutic dose of APAP, and protein-derived APAP-cysteine is detectable after supratherapeutic consumption of APAP without hepatotoxicity (O'Malley et al., 2015). Recently, Roberts et al. (2017) differentiated ALI from the others using APAP protein adducts by developing an immunoassay that rapidly measures APAP protein adducts to identify ALF. Therefore, considering the presence of APAP protein adducts at the therapeutic dose of APAP, formation of protein adducts on mitochondrial proteins rather than the overall formation of protein adducts may be the reason for cellular toxicity, which has been considered a key factor for necrotic cell death in previous years (Jollow et al., 1973; Hu et al., 2016).

Depleted glutathione by NAPQI leads to increased H_2_O_2_ release that oxidizes thioredoxin and induces the disassociation of thioredoxin from apoptosis signalling-regulating kinase 1 (ASK-1), triggering self-activation of ASK-1, which phosphorylates mitogen-activated protein kinase kinase 4/7 and activates c-Jun N-terminal kinase (JNK) (Hanawa et al., 2008; Nakagawa et al., 2008). Activated JNK contributes to the dysfunction of the electron transport chain and increased ROS. In addition, sustained JNK activation was amplifies mitochondrial ROS and forms a self-sustaining activation loop (Win et al., 2016). Furthermore, p-JNK leads to the activation of Bax and translocation to mitochondria to trigger the mitochondrial permeability transition (MPT) pore formation, along with collapsed ATP depletion and membrane potential, eventually causing DNA fragmentation and necrosis (Bajt et al., 2006; Kim et al., 2006). In recent years, Kim et al. (2015) demonstrated that metformin, a first-line drug for treatment of type 2 diabetes mellitus, has therapeutic effects against ALI by inhibiting p-JNK *via* upregulation of Gadd45β. However, a follow-up study showed that protective effect of metformin treatment in APAP-associated hepatotoxicity was independent of JNK (Du et al., 2016). This effect may be associated with other factors, such as the inhibition of ROS production. Therefore, further confirmation of the underlying mechanisms of metformin against APAP-associated hepatotoxicity is necessary. Apart from metformin, p53 (a tumour suppressor protein) is activated due to oxidative stress in ALI, and protective during liver injury *via* inhibition of JNK, but also delays liver regeneration (Huo et al., 2017; Borude et al., 2018). Therefore, p53 seems not an ideal target for ALI considering its double-sided role in ALI pathogenesis.

**Figure 1:**
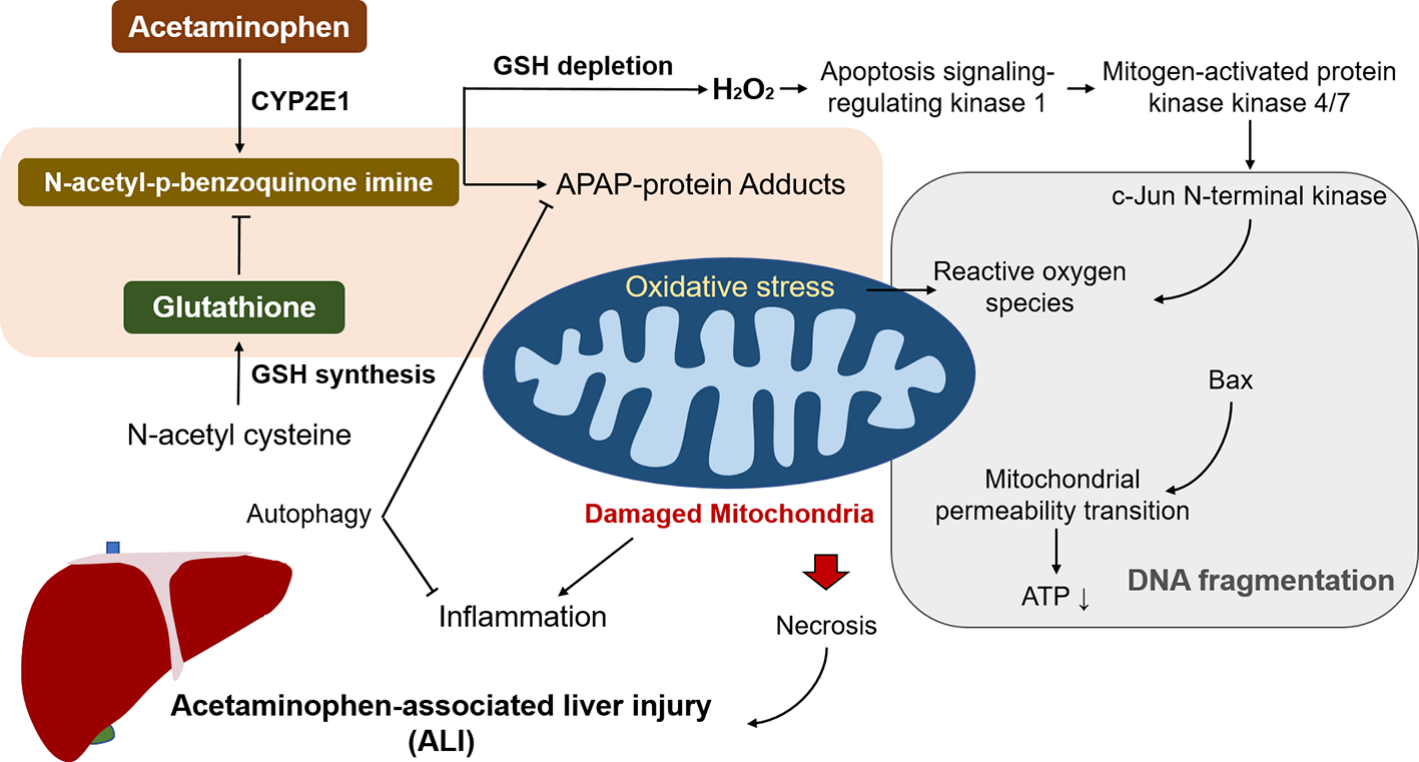
The underlying mechanisms of acetaminophen (APAP) to trigger associated liver injury (ALI). N-acetyl-p-benzoquinone imine generation by APAP overdose leads to depletion of glutathione, giving rise to mitochondrial oxidative stress and damage. It induces ATP depletion and the opening of the mitochondria permeability transition pore that contributes to the mitochondrial protein translocation, resulting in DNA fragmentation and subsequent necrosis. Reactive oxygen species (ROS) caused by N-acetyl-p-benzoquinone imine activates c-Jun N-terminal kinase and the sustained activation triggers mitochondrial ROS, and form a self-sustaining activation loop. Among the processes, autophagy alleviates liver injury by removing damaged mitochondria and detrimental APAP adducts.

Moreover, many other signalling pathways are promoted in response to the oxidative stress in APAP overdose. Nuclear factor erythroid 2-related factor 2 (Nrf2) is notably representative and is activated by NAPQI-induced redox status changes. Nrf2 was found to participate in transcriptional activation of antioxidant enzymes that act as a cell defensive system to detoxify NAPQI (Goldring et al., 2004). In addition to activation by NAPQI, the Nrf2 signalling pathway is also regulated by protein tyrosine phosphatase 1B (PTP1B) and M1 muscarinic receptors (M1Rs) (Mobasher et al., 2013; Urrunaga et al., 2015). APAP-associated hepatotoxicity is ameliorated by promoting the Nrf2 system in mice without PTP1B or M1R, indicating that PTP1B and/or M1R may be promising therapeutic targets in ALI. Considering the positive impact of Nrf2 in ameliorating oxidative stress, a number of bioactive components that exert protective effect against APAP-associated hepatotoxicity have been identified and reported, including caffeic acid that activates the Keap1-Nrf2 antioxidant defense system (Pang et al., 2016), carnosic acid that promotes nuclear translocation of Nrf2 (Guo et al., 2016), and esculentoside A that activates Nrf2 *via* AMP-activated kinase/Akt/glycogen synthase kinase 3 pathway (Wang et al., 2016). These studies reveal that pretreatment with the Nrf2-activating natural products prevents against ALI in APAP overdose. Overall, glutathione depletion, oxidative stress, and mitochondrial damage are the critical underlying mechanisms of ALI (**Figure 1**).

#### Cytochrome P450-Mediated Metabolic Activation

APAP metabolic activation is mainly catalyzed by the cytochrome P450 enzyme and the reactive metabolite associated with hepatotoxicity is commonly considered to be NAPQI (Dahlin et al., 1984). APAP overdose results in excess NAPQI formation that depletes glutathione levels and binds to proteins. A previous study confirmed increased turnover of glutathione after increasing APAP dose (Jollow et al., 1973). Moreover, APAP-protein adducts can be measured in patients with APAP overdose (Davern et al., 2006). In addition, cysteine residues are the main targets for covalent alteration by the reactive intermediate of APAP (Streeter et al., 1984).

**Figure 2:**
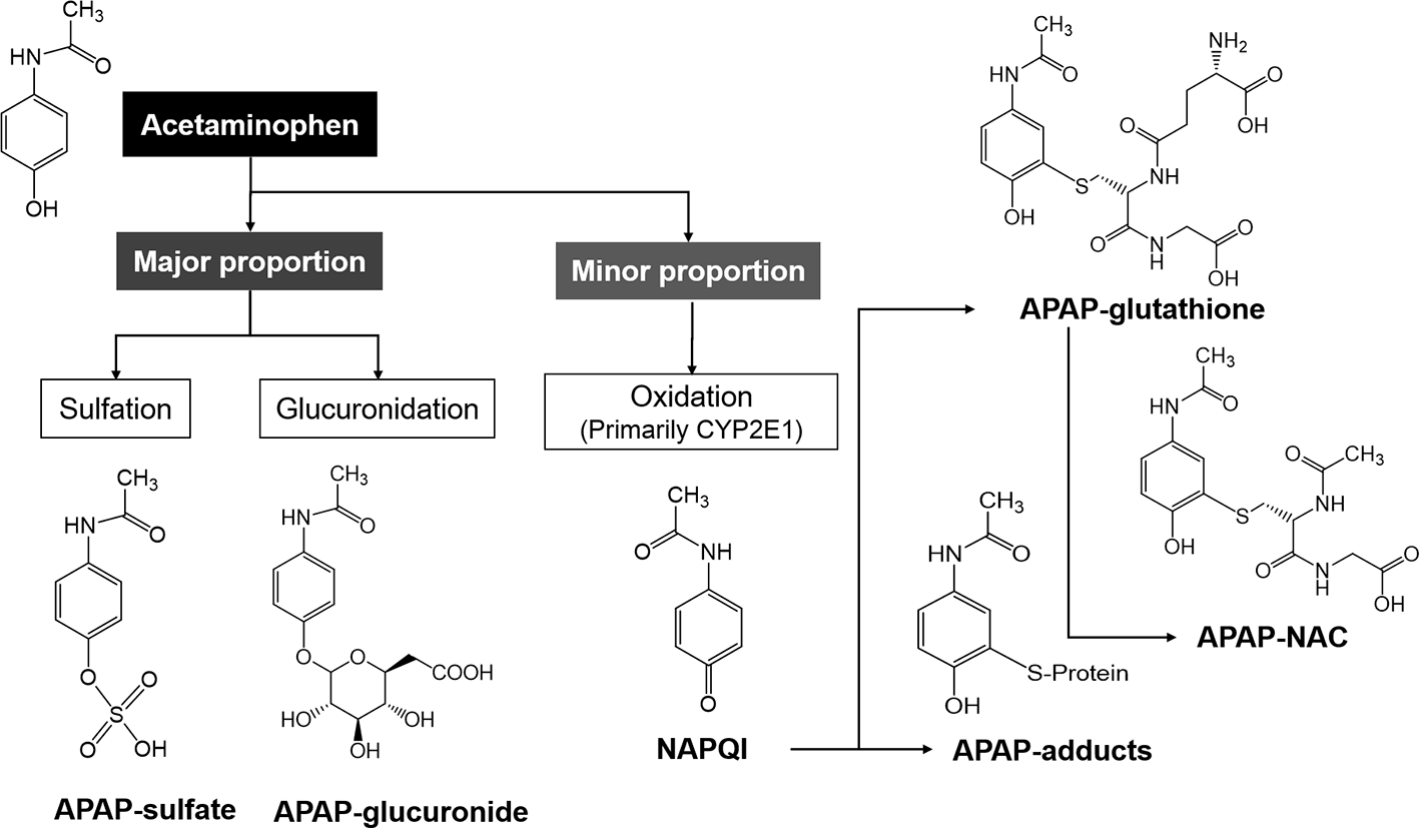
The metabolic pathways of acetaminophen (APAP) in the human body. The major proportion of APAP is mainly metabolized to APAP-sulfate and -glucuronide, and the minor proportion is metabolized by CYP2E1 to NAPQI, which is subsequently metabolized to APAP-glutathione and -NAC.

Both alcohol and isoniazid induce CYP2E1 and affect APAP-induced ALI by increasing NAPQI, the hepatotoxic metabolite of APAP, indicating that CYP2E1 may be the major P450 contributing to conversion of APAP to NAPQI (Zand et al., 1993; Thummel et al., 2000). Accordingly, less susceptibility of alcohol and isoniazid was observed in CYP2E1-knockout mice in an ALI setting (Lee et al., 1996). Apart from CYP2E1, CYP1A2, 2D6, and 3A4 have also been found to activate APAP in various models (Patten et al., 1993; Thummel et al., 1993; Dong et al., 2000). Beta-catenin-deficient mice have been reported to show elimination of Cyp2e1 and 1a2 proteins, which are associated with resistance against APAP hepatotoxicity (Sekine et al., 2006). Moreover, Cyp2e1-/- mice showed resistance to the APAP high dose-associated hepatotoxicity, whereas human CYP2E1 transgenic mice were susceptible, suggesting that CYP2E1 is the major P450 enzyme participating in APAP activation (Cheung et al., 2005). Furthermore, APAP-protein adducts were detected after APAP treatment in human HepaRG cells that express low CYP2E1 levels (Antherieu et al., 2010). Therefore, CYP2E1 is the primary enzyme contributing to the conversion of APAP to its reactive intermediate along with other P450s, such as CYP1A2 and CYP3A4. At normal therapeutic dose, APAP is mainly metabolized *via* O-glucuronidation (50%–70%) and O-sulfation (approximately 25%) pathways and forms the corresponding conjugates that can be easily excreted by the kidney. Moreover, a minor proportion of this agent can be metabolized within the liver by the cytochrome P450 enzymes (primarily by CYP2E1) to NAPQI, which is detoxified by conjugation and excreted in the glutathione or NAC forms (**Figure 2**). However, when the drug is taken in an overdose, the Uridine diphosphate-glucuronosyltransferases (UGTs) and sulfotransferases (SULTs) in the human liver that participate in APAP detoxification become saturated, and a considerable proportion of this drug is activated by human CYPs (primarily CYP2E1).

For the treatment of AALI (APAP-associated ALI), only NAC has been used as a classic therapy for APAP-associated hepatotoxicity induced by NAPQI. In recent years, cimetidine has been used to reduce toxic metabolites by inhibiting cytochrome p450; thus, some researchers have suggested using cimetidine as a complementary treatment for AALI (Al-Mustafa et al., 1997). In addition, cimetidine is an available drug and its side effects could be found at very high doses (Woodhead et al., 2012). However, a double-blinded clinical trial involving 105 patients suspected of APAP toxicity who require medical treatment from Iran. Comparing the therapeutic efficacy of NAC or combination of NAC and cimetidine in patients with acute AALI, no significant improvement was found in hepatoprotective activities between intravenous administration of cimetidine (300 mg per 6 h) and the sole NAC treatment (Ebrahimi et al., 2015). However, the impact of cimetidine for prevention of ALI in individuals using APAP remains to be confirmed in the future.

### APAP-Associated Idiosyncratic DILI

Both APAP-associated intrinsic and idiosyncratic DILI are defined by ALT elevation (≥5 × the upper limits of normal, resulting in difficulty in differentiating one from the other (Teschke and Zhu, 2018). Overdose of APAP (≥15 g) has been reported to contribute to the classic intrinsic DILI, including ALF. However, low-dose use of APAP, including daily doses, may lead to idiosyncratic injury and liver adaptation (Yoon et al., 2016; Ramachandran and Jaeschke, 2018). In a Spanish study evaluating 32 prospectively gathered ALI cases, the APAP-associated Roussel Uclaf Causality Assessment Method (RUCAM) score was higher compared to that of other concomitant medications. The estimated incidence of AALI was 0.4 per million (99% confidence interval [CI], 0.2–0.8) in individuals >15 years of age and 10 per million (95% CI, 4.3–19.4) in APAP users (Sabate et al., 2011). In addition, APAP-associated idiosyncratic DILI was shown to have immunological manifestations, such as arthralgia, fever, eosinophilia, rash, and thrombocytopenia. Taken together, for rapid and precise detection and/or treatment of APAP-associated idiosyncratic DILI, it is necessary to assess the causality by the RUCAM score.

### APAP-Associated Liver Adaptation

Liver adaptation to APAP may occur with therapeutic doses in some individuals. APAP overdose could cause ALF, but in several studies, repeated exposure to APAP led to autoprotective effects (Eakins et al., 2015). A randomized controlled trial identified that the incidence of ALT elevation (>3 × upper limits of normal) was 31% to 44% in treatment groups receiving APAP (Watkins et al., 2006). The median maximum ALT was 2.78 (95% CI, 1.47–4.09; P < 0.001) compared to placebo, suggesting history of APAP ingestion to be considered in differential diagnosis of ALT elevations regardless presence of serum APAP concentrations. Liver adaptation usually presents without clinical symptoms along with relatively low serum liver tests, including ALT that often remain at <5 × the upper limits of normal. Most of liver adaptations recover ALT levels that gradually drop to normal range, and no clinical decision is required. Therefore, even the serum APAP concentration is negative, a slight increase in liver tests without clinical manifestations or the history of APAP ingestion may refer to the liver adaptation.

### Advances in Herbal Therapy for ALI

To date, a number of herbal extracts have been identified as effective for prevention of AALI with reasonable evidence in terms of effectiveness and safety (**Table 1**). In addition, there are also a number of therapeutic approaches using the natural product extracts after APAP administration with significant amelioration of the liver function and improved survival (**Table 2**).

**Table 1:** Effects of bioactive natural product extract pretreatment in ALI.

Herbal	Extract dose	APAP dose	Effect/mechanism	Ref.
*Acacia catechu* (L.f.) Willd.	400 mg/kg	750 mg/kg	LPO accumulation↓Antioxidant	(Lakshmi et al., 2018)
*Adansonia digitata* L.	200 mg/kg	100 mg/kg	ALP, ALT, AST, MDA,SOD↓	(Hanafy et al., 2016)
*Alnus japonica* (Thunb.) Steud.	200 μg/ml	500 mg/kg	Antioxidant	(Kim et al., 2004)
*Arctium lappa* L.	300 mg/kg	800 mg/kg	ALP, ALT, AST, DNA fragmentation↓	(El-Kott and Bin-Meferij, 2015)
*Artemisia absinthium* L.	500 mg/kg	640 mg/kg	MDME↓	(Gilani and Janbaz, 1993)
*Artemisia scoparia* Waldst. & Kitam.	150 mg/kg	640 mg/kg	ALT, AST↓	(Gilani and Janbaz, 1995a)
*Camellia sinensis* (L.) Kuntze (Green tea)	1000 mg/kg	200mg/kg	ALT, AST, ALP, TB↓	(Salminen et al., 2012; Wei et al., 2018)
*Centaurium erythraea* Rafn	900 mg/kg	750 mg/kg	ALT, AST, LDH↓	(Mroueh et al., 2004)
*Cuscuta chinensis* Lam.	250 mg/kg	835 mg/kg	ALP, ALT, AST, SOD, CAT, MDA↓	(Yen et al., 2007)
*Cuscuta campestris* Yunck.	250 mg/kg	850 mg/kg	ALT, AST, SOD, CAT, MDA, GPx↓	(Koca-Caliskan et al., 2018)
*Cyperus scariosus* R.Br.	500 mg/kg	1 g/kg	ALP, ALT, AST↓	(Gilani and Janbaz, 1995b)
*Genista quadriflora* Munby	300 mg/kg	1 g/kg	CYP2E1, GSTpi, TNF-α↓	(Baali et al., 2016)
*Glossocardia bidens* (Retz.) Veldkamp	300 mg/kg	500 mg/kg	ALT, AST↓GSH↑	(Tien et al., 2014)
*Hydrastis canadensis* L.	1000 mg/kg	400 mg/kg	ALT, AST, Cytochrome P450 2E1↓	(Yamaura et al., 2011)
*Musanga cecropioides* R.Br. ex Tedlie	500 mg/kg	800 mg/kg	ALT, AST, OTC↓	(Adeneye, 2009)
*Paeonia × suffruticosa* Andrews	400 mg/kg	400 mg/kg	ALT, GSH, Cytochrome P450 2E1, Hepatic DNA damage↓	(Shon and Nam, 2004)
*Premna tomentosa* Willd.	750 mg/kg	640 mg/kg	Membrane-bound enzymes↓	(Lakshimarayen and Muthana, 1953)
*Prosopis farcta* (Banks & Sol.) J.F.Macbr.	75 mg/kg	600 mg/kg	ALT, AST, TG, LDL, VLDL↓	(Asadollahi et al., 2014)
*Pterocarpus erinaceus* Poir.	75 mg/kg	2 g/kg	ALT, AST, MDA↓GSH↑	(Akinmoladun et al., 2015)
*Sasa veitchii* (Carrière) Rehder	0.2 mL/day	550 mg/kg	ALT, AST, JNK, RIP-1, CYP2E1↓	(Zhang et al., 2014; Yoshioka et al., 2017)
*Schisandra sphenanthera* Rehder & E.H.Wilson	700 mg/kg	400 mg/kg	ALT, AST, MDA↓	(Fan et al., 2014; Fan et al., 2015)
*Teucrium polium* L.	250 mg/kg	500mg/kg	ALT, AST, ALP↓	(Sakeran et al., 2014)
*Trifolium alexandrinum* L.	300 mg/kg	1 g/kg	CYP2E1, GSTpi, TNF-α↓	(Forouzandeh et al., 2013)
*Tournefortia sarmentosa* Lam.	500 mg/kg	1000 mg/kg	MDA↓	(Teng et al., 2012)
*Zingiber officinale* Roscoe	0.3 mL/kg 400 mg/kg	600 mg/kg 3 g/kg	ALP, ALT, AST, LDH, SDH↓ Antioxidant	(Yemitan and Izegbu, 2006) (Ajith et al., 2007)

**Table 2:** Therapeutic effects of bioactive natural product after APAP overdose.

Herbal	Extract dose	APAP dose	Effect	Ref
*Artemisia absinthium* L.	500 mg/kg	640 mg/kg	MDME↓	(Gilani and Janbaz, 1993)
*Baccharis dracunculifolia* DC.	50 mg/kg	600 mg/kg	ALT, AST, ALP	(Guimaraes et al., 2012)
*Camellia sinensis* (L.) Kuntze (Green tea)	1000 mg/kg	150 mg/kg	ALP; ALT; GSH; AST, TB↓	(Salminen et al., 2012; Wei et al., 2018)
*Citrus × aurantium* L.	1000 mg/kg	300mg/kg	Survival rate	(Yamaura et al., 2012)
*Hydrastis canadensis* L.	1000 mg/kg	400 mg/kg	ALT, AST, Cytochrome P450 2E1↓	(Yamaura et al., 2011)
*Moringa oleifera* Lam	400 mg/kg	7 g/kg	GSH, SOD, CAT↓Antioxidant	(Fakurazi et al., 2012; Sharifudin et al., 2013)
*Phyllanthus urinaria* L.	200 mg/kg	550 mg/kg	Cytochrome P450 2E1↓	(Hau et al., 2009)
*Punica granatum* L.	50 mg/kg	750 mg/kg	ALT, AST↓Antioxidant	(Ahmad et al., 2016)
*Tournefortia sarmentosa* Lam	500 mg/kg	1000 mg/kg	ALP, ALT, AST, TNF-α, IL-1β, IL-6, CAT, SOD, GPx↓	(Teng et al., 2012)
*Trifolium alexandrinum* L.	100 mg/kg	100 mg/kg	ALP; ALT, GGTP, TB, MDA↓	(Sakeran et al., 2014)
*Vitex doniana* Sweet	400 mg/kg	300 mg/kg	ALP, ALT, AST, GPx↓	(Ajiboye, 2015)

### *Adansonia digitata* L.

The methanol extract of *Adansonia digitata L*. (Malvaceae), which is a native tree in Central Africa and commonly known as baobab, fruit pulp (the pulp is dissolved in milk or water to be used as a drink or a sauce for food and is considered one of the popular ingredients in ice products) was also found to exert protective effects against ALI (Hanafy et al., 2016). The protection was mediated through attenuating lipid peroxidation by the scavenging activity of free radicals, as well as the antioxidant activities.

### *Alnus japonica* (Thunb.) Steud.

Pretreatment with the stem bark of the Betulaceae plant *Alnus japonica* (Thunb.) Steud., a common folk medicine for cancer and hepatitis indigenous to Korea, revealed dose-dependent antioxidant effects in APAP-associated hepatotoxicity (Kim et al., 2004). The Alnus species are characterized by high amounts of the triterpenoids and diarylheptanoids compounds; thus, it is suggested that phenolic compounds may be associated with the antioxidant effects (Tadashi et al., 1990).

### *Arctium lappa* L.

A Saudi Arabian study have confirmed ameliorative effect of *Arctium lappa* L., which has been used as the tea, root extracts against ALI by confirmation on suppressed malondialdehyde content and mitigated most of the hepatic tissue damages caused by APAP overdose in rats, but some necrotic areas, as well as dilated and congested central vein, remained in Artium lappa extract treatment group even 30 days after APAP treatment (El-Kott and Bin-Meferij, 2015). It is important to note that there was not full recovery from APAP-associated toxicity a month after the treatment.

### *Artemisia scoparia* Waldst. & Kitam. and *Artemisia absinthium* L.

In 1993, hydromethanolic extract of *Artemisia scoparia* Waldst. & Kitam. (Compositae) was first reported to protect liver against APAP-induced hepatotoxicity (Gilani and Janbaz, 1993). Pretreatment of the extract (150 mg/kg) could significantly decrease the mortality by reducing serum alanine aminotransferase (ALT) and aspartate aminotransferase (AST) levels when applied APAP at a dose of 1 g/kg, which was a 100% lethal dose in the experiment. In addition, extract from *Artemisia absinthium* L. pretreated with aqueous-methanolic(Compositae) (500 mg/kg) also had the hepatoprotective effects by reducing ALT and AST levels, and inhibiting microsomal drug-metabolizing enzymes (Gilani and Janbaz, 1995a).

### *Baccharis dracunculifolia* DC.

Glycolic extract of *Baccharis dracunculifolia* DC. that contains several chemical compounds, including caffeic acid, p-coumaric acid, cinnamic acid, aromadendrin, isosakuranetin, and artepillin C, was previously reported as the main source of green propolis with potent antioxidant activity that prevents against oxidative mitochondrial damage (Guimaraes et al., 2012). The extract of the leaves was testified in APAP-induced hepatotoxicity that revealed high total phenolic and flavonoid contents, which are hepatoprotective properties (Rezende et al., 2014).

### *Brassica juncea* (L.) Czern. and *Angelica keiskei* (Miq.) Koidz.

*Brassica juncea* (L.) Czern. (Brassicaceae; Indian mustard) is considered to possess diverse pharmacological properties. The mustard seed extract was testified for APAP-induced toxicity in a hepatocellular carcinoma cell line (HepG2) and was found to have protective effects in both post and pretreatment models with a 20 mM APAP dose (Parikh et al., 2015). Phytochemical analysis showed the presence of vitamin, tannin, flavonoid, and phenolics, and the antioxidant activity revealed a linear correlation with the contents. However, the extent of ROS generation significantly differed that posttreatment inhibited the ROS generation by 58.4%, whereas pretreatment suppressed the generation of ROS by 90.5%. Further experiments confirmed the presence of vitamin E, quercetin, and cetechin, which demonstrated hepatoprotective effect, and the ROS inhibition was confirmed to be associated with antioxidant activities, thus protecting against ALI. An ethanol extract of *Angelica keiskei* (Miq.) Koidz. was also testified in HepG2 and HepaRG cells by culturing with 30 mM APAP (Choi et al., 2017). The extract was found to downregulate apoptotic factors, such as caspase-3, -7, and -9, *via* intrinsic and extrinsic pathways to prevent ALI in APAP-induced hepatotoxicity.

### *Camellia sinensis* (L.) Kuntze

Green tea comes from *Camellia sinensis* (L.) Kuntze. Salminen et al. (2012) tested the effect of green tea extract prior to and 6 h after APAP administration. Independently verified key components of the green tea extract batch GTE50-A0302031114 were polyphenols, catechins, gallates, caffeine, moisture, ash, heavy metals and residues, and these results suggest that green tea extract compounds may downregulate or suppress CYP1A2, 2E1, and 3A4 reduce APAP conversion to the reactive metabolite NAPQI. Interestingly, pretreatment of green tea extract revealed to provide protection against AALI by decreasing APAP covalent binding to protein, thus relatively reducing reactive metabolites for liver injury. However, green tea extract potentiated APAP-induced hepatotoxicity when applied after APAP treatment through causing glutathione depletion, indicating importance of the supplement interactions. Recently, Wei et al. (2018) found that the natural polyphenol chlorogenic acid is protective against APAP-induced hepatotoxicity through activation of Nrf2 antioxidant signalling pathway by blocking the binding of Nrf2 to Keap1. In addition, ERK1/2 plays a critical role in regulation of the polyphenol chlorogenic acid-derived Nrf2 transcriptional activation. These findings were further supported by a study from the United States that also confirmed increases in serum transaminases and histopathologic scores when administered the green tea extract after APAP (Lu et al., 2013).

### *Centaurium erythraea* Rafn

The methanol extract of the leaves from *Centaurium erythraea* Rafn (Gentianaceae; 300 mg/kg per day for 6 days or 900 mg/kg for one day) was found the hepatoprotective effect against APAP-induced hepatotoxicity by reducing the serum concentration of ALT, AST, and lactate dehydrogenase (LDH) in patients (Mroueh et al., 2004).

### *Citrus* × *aurantium* L.

The extract of a citrus fruit (natsumikan; Citrus × aurantium L.) that contains antioxidant nutrients, such as vitamin C and flavonodis, was found effective in improving survival of mice against acute ALI (Yamaura et al., 2012). Administration of 300 mg/kg of APAP led to 100% mortality within 6 h, but 1,000 mg/kg of natsumikan extract and silymarin prolonged survival rates to 33.3% and 50.0%, respectively.

### *Cuscuta chinensis* Lam. and *Cuscuta campestris* Yunck.

The dual effects of ethanolic and aqueous extracts of the seeds of *Cuscuta chinensis* Lam. (Convolvulaceae), a traditional Chinese medicine used to nourish the liver and kidney, were noticed in APAP-induced hepatotoxicity (Yen et al., 2007). The ethanolic extract prevented the hepatotoxicity induced by APAP and had significant antioxidant activity, but the same dose of aqueous extract showed no hepatoprotective effect, and led to further deterioration of the liver, suggesting importance of the extract types. Moreover, the identified pharmacologically active constituents of the Cuscuta chinensis include polysaccharide, lignans, quinic acid, flavonol, and flavonoids (Du et al., 1998; Wang et al., 2000; Ye et al., 2002). Additionally, Yen et al. (2008) testified that the nanoparticle formulation of *Cuscuta chinensis* Lam. was superior to ethanolic extract/aqueous extract in terms of overcoming water-poorly-soluble and the decreasing the treatment dose. Furthermore, the aqueous and methanolic extracts of *Cuscuta campestris* Yunck. (125 and 250 mg/kg), a parasitic plant commonly known as dodder that is used as a folk medicine to treat liver-related diseases, were found to notably exert hepatoprotective and antioxidant effects (Koca-Caliskan et al., 2018).

### *Cyperus scariosus* R.Br.

Further examination revealed that *Cyperus scariosus* R.Br. (Cyperaceae) extract (500 mg/kg) had a hepatoprotective effect confirmed by decreased the levels of ALT, AST, and alkaline phosphatase (ALP) in APAP overdose patients/mice (Gilani and Janbaz, 1995b).

### *Genista quadriflora* Munby and *Teucrium polium* L.

Two of the endemic plants (*Genista quadriflora* Munby and *Teucrium polium* L.) in France that are frequently used for dietary and/or medical applications were testified and compared for its hepatoprotective effectiveness against APAP-induced hepatotoxicity in rats (Baali et al., 2016). Pretreatments of the rich-polyphenol fractions of them (300 mg/kg per day for 10 days) prior to oral administration of APAP (1 g/kg) exerted a hepatoprotective effect against ALI by improving transaminase leakage and enhancing antioxidant defense. In addition, suppressed miRNA expression of CYP2E1, GSTpi, and TNF-α along with enhanced mitochondrial bioenergetics were suggested to be responsible for the protective effect. It was further suggested that predominant flavonoids and phenolic acids in the extracts may have contributed to the high total polyphenol content.

### *Glossocardia bidens* (Retz.) Veldkamp

In BALB/c mice, hot water extracts of *Glossocardia bidens* (Retz.) Veldkamp was found to contain high phenolics (chlorogenic acid and luteolin-7-glucoside). The extracts were effective at protecting against ALI by increasing glutathione levels, reducing the ratio of glutathione to oxidized glutathione in the liver, and inhibiting peroxidation to exert antioxidant ability (Tien et al., 2014). In addition, the antioxidant capacity of *Glossocardia bidens* (Retz.) Veldkamp was dependent on the type of the solvents, including hot water, 50% ethanol, and 95% ethanol. It is crucial to note that the capacity was most excellent when applied hot water.

### *Hovenia Dulcis* Thunb.

In China, although *Hovenia Dulcis* Thunb is frequently used to protect the liver against alcoholic injury, its impact against APAP-induced hepatotoxicity remained unclear. Dong et al. (2018) have confirmed hepatoprotective effects of an ethanol extract of Hovenia Dulcis against ALI in a dose-dependent manner by suppressing cytochrome P450 activity, cell apoptosis, and modulation of bile acid homeostasis imbalance.

### *Hydrastis canadensis* L.

Goldenseal (*Hydrastis canadensis* L.) was further confirmed to be protective against AALI by inhibiting CYP2E1 in rats (Yamaura et al., 2011). Moreover, goldenseal can also inhibit the cytochrome P450 isoforms, including CYP1A2, CYP2D6, CYP2E1, and CYP3A, and the IC_50_ values were 15.7, 7.4, 4.3, and 52.1 µg/ml in rats, respectively.

### *Moringa oleifera* Lam.

Fakurazi et al. (2012) found that the administration (1 h after APAP) of extract from *Moringa oleifera* Lam. (Moringaceae), an antioxidant with abundant essential minerals, was significantly effective in reducing malondialdehyde and 4-hydroxynonenal protein adduct levels. In addition, the Folin-Ciocalteu assay of the total phenolic compounds revealed that the ethanolic extract of Moringa oleifera has the highest total phenolic content (24.21 ± 1.55 mg gallic acid equivalent/100 g dw followed by leaves, pods, seeds, and stem. Further research confirmed that administration of the extract alleviated the severity of liver injury (Sharifudin et al., 2013).

### *Musanga cecropioides* R.Br. ex Tedlie and *Vitex doniana* Sweet

Pretreatment with aqueous extract of the stem bark *Musanga cecropioides* R.Br. ex Tedlie (125–500 mg/kg), which is used as a natural antidote for gastric poisoning in Southwest Nigeria, significantly ameliorated acute elevation of the liver enzymes and hepatotoxicity-associated histopathological lesions within the liver by activating natural antioxidants (flavonoid and alkaloid) (Adeneye, 2009). The extract of another local Nigerian remedy (*Vitex doniana* Sweet) for the treatment of liver-related diseases was also assessed in APAP-induced hepatotoxicity (Ajiboye, 2015). GC-MS profiling on methanolic extract of Vitex doniana fruits indicated 13 different phytoconstituents with β-sitosterol, which was found to have antioxidant and antiradical activities, as the most abundant compound, followed by platycodin D, apigenin, saikosaponinn, and chrysin. The extract significantly attenuated serum liver enzymes and ameliorated the reduction in superoxide dismutase, catalase, glutathione peroxidase and reductase, and 6-phosphate dehydrogenase activities. Furthermore, the increased conjugated dienes, lipid hydroperoxides, and fragmented DNA levels were significantly decreased when administered methanolic extract of *Vitex doniana* Sweet.

### *Paeonia* × *suffruticosa* Andrews

In the early 21st century, Shon et al. (2004) explored the impact of cortex of *Paeonia × suffruticosa* Andrews and its underlying mechanisms. Administration of cortex of *Paeonia × suffruticosa* Andrews could prevent liver injury by decreasing ALT, protecting against hepatic glutathione depletion, attenuating cytochrome P450 2E1 activity, and preventing hepatic DNA damage *in vivo*.

### *Passiflora subpeltata* Ortega

According to a study from Brazil, luteolin and quercetin 3-β-d-glucoside were newly detected as compounds in *Passiflora subpeltata* Ortega leaves, and an acetone extract of this plant was found to elevate the serum white blood cell, red blood cell, and hemoglobin counts, and retain the serum biochemical and antioxidant levels to normal range, providing protection against ALI (Shanmugam et al., 2016).

### *Phyllanthus urinaria* L.

Oral administration of *Phyllanthus urinaria* L. extract following intraperitoneal administration with a lethal dose of APAP was found to be protective against APAP-induced necrosis by downregulating the cytochrome P450 CYP2E1 protein levels *in vitro* (Hau et al., 2009). In addition, a therapeutic dose of *Phyllanthus urinaria* showed no toxicological phenomena in mice.

### *Premna tomentosa* Willd.

Pretreatment with an extract from Indian traditional medicine *Premna tomentosa* Willd. (Lamiaceae) (750 mg/kg for 15 days) before APAP administration (640 mg/kg). It was found that the Indian traditional medicine could prevent the liver injury-induced membrane damage, suggesting the presence of antioxidant compound limonene (Devi et al., 2004). In addition, previous preliminary phytochemical screening confirmed the presence of limonene (57.8%) along with other phytoconstituents, including beta-caryophyllene (17.2%), cadalene type sesquiterpene (7.8%), ses quiterpene tertiary alcohol (5.6%), and aditerpene (5.5%) (Lakshimarayen and Muthana, 1953).

### *Prosopis farcta* (Banks & Sol.) J.F.Macbr.

Extract of *Prosopis farcta* (Banks & Sol.) J.F.Macbr. beans were previously found to contain a number of antioxidant phenolic compounds, such as vicenin-2, vitexin, glucoside, and luteolin. In Wister albino rats weighing approximately 200 g, 600 mg/kg of APAP administration significantly increased ALT, AST, cholesterol, and low-density lipoprotein, which were attenuated to near normal ranges when administrated 50 and 75 mg/kg of *Prosopis farcta* (Banks & Sol.) J.F.Macbr. beans. Administration of bean extract prior to APAP resulted in hepatoprotective activity against ALI despite relatively low doses (Asadollahi et al., 2014).

### *Pterocarpus erinaceus* Poir., Bilberries, and Blackcurrants

Akinmoladun and colleagues explored the hepatoprotective effects of pretreatments of the ethanol stem bark extract and the constituent flavonoid (homopterocarpin) against APAP (2 g/kg). It was found that these substances ameliorated APAP-associated biochemical alterations. Furthermore, higher antioxidant potentials potential was found in homopterocarpin compared to the ethanol stem bark extract, suggesting the importance of the extract types in preventive efficacy against ALI (Akinmoladun et al., 2015). In addition, an anthocyanin-risk extract from bilberries and black currants was also found to be protective against acute ALI in rats (Cristani et al., 2016).

### *Punica granatum* L.

Surprisingly, 14 days of treatment with *Punica granatum* L. methanolic extract was reported to have very strong effects in ameliorating liver functions after APAP treatment (750 mg/kg) potentially by antioxidant properties that the mean liver ALT and AST levels were even lower in the pomegranate peel posttreatment group after APAP treatment (ALT, 49.6 ± 12.1; AST, 96.1 ± 18.0) compared to the normal saline group without APAP treatment (ALT, 51.5 ± 15.4; AST, 97.9 ± 19.5) (Ahmad et al., 2016).

### *Sasa veitchii* (Carrière) Rehder and *Acacia catechu* (L.f.) Willd.

In accordance with other extracts from natural products, 7 days of a *Sasa veitchii* (Carrière) Rehder leaf extract (0.2 ml per day) pretreatment significantly decreased hepatic injury markers, including ALT and AST, oxidative stresses, including malondialdehyde and glutathione, histologic damages, inflammatory cytokines, activation of JNK, receptor-interacting protein-1, which has emerged as a key modulator of necrotic cell death, and CYP2E1, whereas the total antioxidant capacity within the liver was increased in an acute APAP mice model (550 mg/kg) (Zhang et al., 2014; Yoshioka et al., 2017). More recently, the seed and bark extracts (400 mg/kg) of *Acacia catechu* (L.f.) Willd. were also found to modulate oxidative stress, antioxidative activities, and liver function to protect against APAP-induced hepatotoxicity in rats with higher potentials in the seed extract for protection of the liver (Lakshmi et al., 2018).

### *Schisandra sphenanthera* Rehder & E.H.Wilson and *Schisandra chinensis* (Turcz.) Baill.

In many regions, *Schisandra sphenanthera* Rehder & E.H.Wilson is widely used to support liver function and is applied in the form of Wuzhi tablet, which is composed of ethanol extracts. Administration of Wuzhi tablet 3 days prior to APAP treatment could significantly alleviate hepatoxicity in a dose-dependent manner along with reduction in the activation of JNK (Fan et al., 2014). In addition, potent inhibition of the activities of CYP2E1, CYP3A11, CYP1A2, and KEAP1, and the formation of the oxidized APAP metabolite NAPQI-reduced glutathione, whereas Nrf2 expression was increased. Moreover, pretreatment with Wuzhi tablet suppressed the p53/p21 signalling pathway, which induces cell proliferation proteins that enhance proliferation of hepatocytes. More recently, they compared therapeutic efficacy of Wuzhi tablet to N-acetylcysteine, which is the main antidote for APAP toxicity (Fan et al., 2015). When administered Wuzhi tablet 4 h after APAP treatment, it revealed much better therapeutic impact compared to N-acetylcysteine, which were confirmed by morphological, histological, and biochemical evaluations. In addition, Wuzhi tablet additionally stimulated liver regeneration after injury, which were verified by increased expressions of cyclin D1, proliferating cell nuclear antigen, and liver regeneration augmenters. Recently, the extracts of *Schisandra chinensis* (Turcz.) Baill. stems revealed to exert significant amelioration in APAP-induced apoptosis, inflammation, and oxidative stress through regulation of mitogen-activated protein kinase and caspase-3 signaling pathway (Li et al., 2018).

### *Tournefortia sarmentosa* Lam.

APAP induces hepatotoxicity, attenuates lipid peroxidation, and enhances antioxidant enzyme activation, including increases in interleukin (IL)-1β, IL-6, and tumor necrosis factor-α elevated (Yen et al., 2008). Pretreatment with the extract of another Chinese herbal medicine, [*Tournefortia sarmentosa* Lam. (Boraginaceae)] which is commonly used as an antiinflammatory or detoxicant agent, showed significantly reduced serum concentrations of ALT, AST, ALP, and inflammatory markers (Teng et al., 2012).

### *Trifolium alexandrinum* L. and *Teucrium polium* L.

According to Sakeran et al. (2014) compared the APAP group to the APAP plus *Trifolium alexandrinum* L. root extract group, the extract improved ALI in terms of histopathology and DNA fragmentation. Pretreatment of *Teucrium polium* L. (Labiatae) extract was also found to be protective against ALI at doses of 250 and 500 mg/kg as confirmed by histological examinations (Forouzandeh et al., 2013).

### *Zingiber officinale* Roscoe

Ethanol extract of the rhizome of *Zingiber officinale* Roscoe was effective in ameliorating liver function as confirmed by attenuated serum ALT, AST, ALP, LDH, and succinate dehydrogenase (Yemitan and Izegbu, 2006). *Zingiber officinale* Roscoe also prevented APAP-induced acute hepatotoxicity by preventing the decline of liver antioxidant status or on account of the radical scavenging capacity (Ajith et al., 2007). Because of the crucial involvement of oxidative damage in AALI, antioxidant properties were further tested in AALI.

## Future Perspectives and Conclusions

Since the early 1990s, numerous extracts from various natural products have been identified as hepatoprotective at different doses against APAP-induced toxicity by ameliorating oxidative stress and liver enzymes. Despite a large amount of effort and many publications, most studies have testified effects of the extract pretreatment prior to acute APAP administration on serologic, histologic, and morphologic liver-related outcomes *in vivo*, but few studies have explored these effects in hepatic cell lines by culturing with the natural products and administrating APAP. In addition, some of the products contained compounds even more effective than the antidote (NAC) when treated after APAP administration. Few studies have compared these highly effective products, and it is still a challenge to determine the greatest potential products. Therefore, it seems to us that only the number of hepatoprotective products has increased without clinical relativity. Recently, gut microbiota was identified to play a key role in the diurnal variation of APAP-induced hepatotoxicity (Gong et al., 2018). Accumulating evidence implicates herbals could treat disease by regulating the gut microbiota (Tong et al., 2018; Anlu et al., 2019; Feng et al., 2019). More studies are needed to verify whether the hepatoprotective effect of natural products through this novel mechanism. In addition, further identifications of the following factors may support prevention and treatment of ALI: specific inhibitors of CYPs such as CYP2E1; induction of detoxifying enzymes (UGT, SULT, etc.) that participate in APAP metabolism (Wang et al., 2015; Cao et al., 2017); treatments to increase or supplement of GSH or NAC to inactivate the reactive metabolite NAPQI rapidly; factors associated with reducing formations of oxidative stress and oxygen free radicals, and protective and preventive factors against mitochondrial damage since mitochondrial dysfunction and damage plays pivotal role in APAP-associated ALI (Shan et al., 2018). As is known in China and other Asian countries, herbal medicines have been widely used in clinical settings. In order to ensure the efficacy and repeatability in herbal medicines, the Chinese government has required all manufacturers to formulate strict quality standards for each herb product. Currently, the majority of herbal medicines used in the clinical settings are produced by several large pharmaceutical companies rather than traditional decocting modes. In fact, part of the herbal medicines used for alleviating ALI are granules of a single herb or several herbs, their major constituents have been reported previously. However, herbal therapy for APAP-induced liver injury remains limited in terms of clinical application due to prospective validation by clinical trials. In addition, individual compounds of the herbal extracts are barely studied even in experimental animal models.

The potential hepatotoxicity of herbal products has also been recognized in recent years (Stickel and Shouval, 2015). Many herbal products have been reported by numerous studies to cause liver injury. The clinical symptoms ranging from acute chronic hepatitis to ALF (Frenzel and Teschke, 2016; Zhu et al., 2019). Although oxidative stress and necrosis were reported to be related to herbal products toxicities, the underlying mechanism is largely unknown (Stickel and Shouval, 2015). More studies are needed to explore the mechanism of natural products side effect. Due to their multicomponent nature and multitargeted and synergistic effects, it is extremely challenging to study the specific molecular mechanism of herbal medicine to alleviate APAP-induced liver injury. In future, the researchers may use the information listed in this review as a clue to deeply understand the bioactive compounds in these herbal medicines for alleviating APAP-induced hepatoxicity, and their mechanisms of action. Considering the preventive, protective, and therapeutic effectiveness of the herbal therapies, evaluation of efficacy and safety in clinical trials may also be promising in the future after further confirming effects of each extract type, interactions between the supplements, and synergism between products.

## Author Contributions

LC, DX, and JZ: design of the study and writing of the manuscript. GG, XK, and YZ: revision of the manuscript and final approval.

## Funding

This work was supported by Shanghai Pudong Commission of Health and Family Planning (PWRd2016-12) to YZ, Talents Training Program of the Seventh People’s Hospital, Shanghai University of Traditional Chinese Medicine (XX2020-19) to LC; National Natural Science Foundation of China (81500494 and 81873590) to JZ, National Natural Science Foundation of China (81922070), Shanghai City Youth Science and Technology Star Project (19QA1405500) to JZ; Shanghai City Youth Science and Technology Yangfan Project (19YF1428200) to DX; National Natural Science Foundation of China (81670562) to XK.

## Conflict of Interest

The authors declare that the research was conducted in the absence of any commercial or financial relationships that could be construed as a potential conflict of interest.

## References

[B1] Adeneye A. A. (2009). Protective activity of the stem bark aqueous extract of Musanga cecropioides in carbon tetrachloride- and acetaminophen-induced acute hepatotoxicity in rats. Afr. J. Tradit. Complement Altern. Med. 6 (2), 131–138. 10.4314/ajtcam.v6i2.57084 20209004 PMC2816566

[B2] Ahmad N. Tahir M. Lone K. P. (2016). Amelioration of acetaminophen induced hepatotoxicity by methanolic extract of pomegranate peels in rats. J. Pak. Med. Assoc. 66 (7), 859–863. 27427136

[B3] Ajiboye T. O. (2015). Standardized extract of Vitex doniana Sweet stalls protein oxidation, lipid peroxidation and DNA fragmention in acetaminophen-induced hepatotoxicity. J. Ethnopharmacol. 164, 273–282. 10.1016/j.jep.2015.01.026 25645189

[B4] Ajith T. A. Hema U. Aswathy M. S. (2007). Zingiber officinale Roscoe prevents acetaminophen-induced acute hepatotoxicity by enhancing hepatic antioxidant status. Food Chem. Toxicol. 45 (11), 2267–2272. 10.1016/j.fct.2007.06.001 17637489

[B5] Akinmoladun A. C. Olaleye M. T. Komolafe K. Adetuyi A. O. Akindahunsi A. A. (2015). Effect of homopterocarpin, an isoflavonoid from Pterocarpus erinaceus, on indices of liver injury and oxidative stress in acetaminophen-provoked hepatotoxicity. J. Basic Clin. Physiol. Pharmacol. 26 (6), 555–562. 10.1515/jbcpp-2014-0095 25811665

[B6] Al-Mustafa Z. H. Al-Ali A. K. Qaw F. S. Abdul-Cader Z. (1997). Cimetidine enhances the hepatoprotective action of N-acetylcysteine in mice treated with toxic doses of paracetamol. Toxicology 121 (3), 223–228. 10.1016/s0300-483x(97)00069-3 9231700

[B7] Aminoshariae A. Khan A. (2015). Acetaminophen: old drug, new issues. J. Endod. 41 (5), 588–593. 10.1016/j.joen.2015.01.024 25732401

[B8] Anlu W. Dongcheng C. He Z. Qiuyi L. Yan Z. Yu Q. (2019). Using herbal medicine to target the “microbiota-metabolism-immunity” axis as possible therapy for cardiovascular disease. Pharmacol. Res. 142, 205–222. 10.1016/j.phrs.2019.02.018 30794922

[B9] Antherieu S. Chesne C. Li R. Camus S. Lahoz A. Picazo L. (2010). Stable expression, activity, and inducibility of cytochromes P450 in differentiated HepaRG cells. Drug Metab. Dispos. 38 (3), 516–525. 10.1124/dmd.109.030197 20019244

[B10] Asadollahi A. Sarir H. Omidi A. Torbati M. B. (2014). Hepatoprotective Potential of Prosopis farcta Beans Extracts against Acetaminophen-induced Hepatotoxicity in Wister Rats. Int. J. Prev. Med. 5 (10), 1281–1285. 25400887 PMC4223948

[B11] Baali N. Belloum Z. Baali S. Chabi B. Pessemesse L. Fouret G. (2016). Protective Activity of Total Polyphenols from Genista quadriflora Munby and Teucrium polium geyrii Maire in Acetaminophen-Induced Hepatotoxicity in Rats. Nutrients 8 (4), 193. 10.3390/nu8040193 27043622 PMC4848662

[B12] Bajt M. L. Cover C. Lemasters J. J. Jaeschke H. (2006). Nuclear translocation of endonuclease G and apoptosis-inducing factor during acetaminophen-induced liver cell injury. Toxicol. Sci. 94 (1), 217–225. 10.1093/toxsci/kfl077 16896059

[B13] Borude P. Bhushan B. Gunewardena S. Akakpo J. Jaeschke H. Apte U. (2018). Pleiotropic Role of p53 in Injury and Liver Regeneration after Acetaminophen Overdose. Am. J. Pathol. 188 (6), 1406–1418. 10.1016/j.ajpath.2018.03.006 29654721 PMC5971235

[B14] Bunchorntavakul C. Reddy K. R. (2018). Acetaminophen (APAP or N-Acetyl-p-Aminophenol) and Acute Liver Failure. Clin. Liver Dis. 22 (2), 325–346. 10.1016/j.cld.2018.01.007 29605069

[B15] Cao L. Greenblatt D. J. Kwara A. (2017). Inhibitory Effects of Selected Antituberculosis Drugs on Common Human Hepatic Cytochrome P450 and UDP-glucuronosyltransferase Enzymes. Drug Metab. Dispos. 45 (9), 1035–1043. 10.1124/dmd.117.076034 28663285 PMC5554070

[B16] Chen Y. H. Lin F. Y. Liu P. L. Huang Y. T. Chiu J. H. Chang Y. C. (2009). Antioxidative and hepatoprotective effects of magnolol on acetaminophen-induced liver damage in rats. Arch. Pharm. Res. 32 (2), 221–228. 10.1007/s12272-009-1139-8 19280152

[B17] Cheung C. Yu A. M. Ward J. M. Krausz K. W. Akiyama T. E. Feigenbaum L. (2005). The cyp2e1-humanized transgenic mouse: role of cyp2e1 in acetaminophen hepatotoxicity. Drug Metab. Dispos. 33 (3), 449–457. 10.1124/dmd.104.002402 15576447

[B18] Choi Y. H. Lee H. S. Chung C. K. Kim E. J. Kang I. J. (2017). Protective effects of an ethanol extract of Angelica keiskei against acetaminophen-induced hepatotoxicity in HepG2 and HepaRG cells. Nutr. Res. Pract. 11 (2), 97–104. 10.4162/nrp.2017.11.2.97 28386382 PMC5376537

[B19] Craig D. G. Lee A. Hayes P. C. Simpson K. J. (2010). Review article: the current management of acute liver failure. Aliment. Pharmacol. Ther. 31 (3), 345–358. 10.1111/j.1365-2036.2009.04175.x 19845566

[B20] Cristani M. Speciale A. Mancari F. Arcoraci T. Ferrari D. Fratantonio D. (2016). Protective activity of an anthocyanin-rich extract from bilberries and blackcurrants on acute acetaminophen-induced hepatotoxicity in rats. Natural Prod. Res. 30 (24), 2845–2849. 10.1080/14786419.2016.1160235 26998559

[B21] Dahlin D. C. Miwa G. T. Lu A. Y. Nelson S. D. (1984). N-acetyl-p-benzoquinone imine: a cytochrome P-450-mediated oxidation product of acetaminophen. Proc. Natl. Acad. Sci. U. S. A 81 (5), 1327–1331. 10.1073/pnas.81.5.1327 6424115 PMC344826

[B22] Davern T. J. 2nd James L. P. Hinson J. A. Polson J. Larson A. M. Fontana R. J. (2006). Measurement of serum acetaminophen-protein adducts in patients with acute liver failure. Gastroenterology 130 (3), 687–694. 10.1053/j.gastro.2006.01.033 16530510

[B23] Devi K. P. Sreepriya M. Balakrishna K. Devaki T. (2004). Protective effect of Premna tomentosa (L. Verbenaceae) extract on membrane-bound phosphatases and inorganic cations transport in acetaminophen-induced hepatotoxicity rats. J. Ethnopharmacol. 93 (2-3), 371–375. 10.1016/j.jep.2004.04.010 15234779

[B24] Dong H. Haining R. L. Thummel K. E. Rettie A. E. Nelson S. D. (2000). Involvement of human cytochrome P450 2D6 in the bioactivation of acetaminophen. Drug Metab. Dispos. 28 (12), 1397–1400. 0090-9556/00/2812-1397–1400 11095574

[B25] Dong S. Ji J. Zhang B. Hu L. Cui X. Wang H. (2018). Protective effects and possible molecular mechanism of Hovenia dulcis Thunb. extract on acetaminophen-induced hepatotoxicity. Pharmazie 73 (11), 666–670. 10.1691/ph.2018.8650 30396387

[B26] Du X. M. Kohinata K. Kawasaki T. Guo Y. T. Miyahara K. (1998). Components of the ether-insoluble resin glycoside-like fraction from Cuscuta chinensis. Phytochemistry 48 (5), 843–850. 10.1016/s0031-9422(97)00990-4 9664709

[B27] Du K. Ramachandran A. Jaeschke H. (2016). Oxidative stress during acetaminophen hepatotoxicity: Sources, pathophysiological role and therapeutic potential. Redox Biol. 10, 148–156. 10.1016/j.redox.2016.10.001 27744120 PMC5065645

[B28] Du K. Ramachandran A. Weemhoff J. L. Chavan H. Xie Y. Krishnamurthy P. (2016). Editor’s Highlight: Metformin Protects Against Acetaminophen Hepatotoxicity by Attenuation of Mitochondrial Oxidant Stress and Dysfunction. Toxicol. Sci. 154 (2), 214–226. 10.1093/toxsci/kfw158 27562556 PMC5139063

[B29] Eakins R. Walsh J. Randle L. Jenkins R. E. Schuppe-Koistinen I. Rowe C. (2015). Adaptation to acetaminophen exposure elicits major changes in expression and distribution of the hepatic proteome. Sci. Rep. 5, 16423. 10.1038/srep16423 26607827 PMC4660393

[B30] Ebrahimi M. Mousavi S. R. Toussi A. G. Reihani H. Bagherian F. (2015). Comparing the Therapeutic Effectiveness of N-acetylcysteine with the Combination of N-acetyl Cysteine and Cimetidine in Acute Acetaminophen Toxicity: A Double-Blinded Clinical Trial. Electron. Physician 7 (6), 1310–1317. 10.14661/1310 26516435 PMC4623788

[B31] El-Kott A. F. Bin-Meferij M. M. (2015). Use of Arctium lappa Extract Against Acetaminophen-Induced Hepatotoxicity in Rats. Curr. Ther. Res. Clin. Exp. 77, 73–78. 10.1016/j.curtheres.2015.05.001 26543508 PMC4564434

[B32] Fakurazi S. Sharifudin S. A. Arulselvan P. (2012). Moringa oleifera hydroethanolic extracts effectively alleviate acetaminophen-induced hepatotoxicity in experimental rats through their antioxidant nature. Mol. (Basel Switzerland) 17 (7), 8334–8350. 10.3390/molecules17078334 PMC6268890 22781444

[B33] Fan X. Jiang Y. Wang Y. Tan H. Zeng H. Wang Y. (2014). Wuzhi tablet (Schisandra Sphenanthera extract) protects against acetaminophen-induced hepatotoxicity by inhibition of CYP-mediated bioactivation and regulation of NRF2-ARE and p53/p21 pathways. Drug Metab. Dispos. 42 (12), 1982–1990. 10.1124/dmd.114.059535 25217484 PMC6067381

[B34] Fan X. Chen P. Jiang Y. Wang Y. Tan H. Zeng H. (2015). Therapeutic efficacy of Wuzhi tablet (Schisandra sphenanthera Extract) on acetaminophen-induced hepatotoxicity through a mechanism distinct from N-acetylcysteine. Drug Metab. Dispos. 43 (3), 317–324. 10.1124/dmd.114.062067 25534769 PMC6067383

[B35] Feng W. Ao H. Peng C. Yan D. (2019). Gut microbiota, a new frontier to understand traditional Chinese medicines. Pharmacol. Res. 142, 176–191. 10.1016/j.phrs.2019.02.024 30818043

[B36] Forouzandeh H. Azemi M. E. Rashidi I. Goudarzi M. Kalantari H. (2013). Study of the Protective Effect of Teucrium polium L. Extract on Acetaminophen-Induced Hepatotoxicity in Mice. Iran. J. Pharm. Res. IJPR 12 (1), 123–129. 24250580 PMC3813216

[B37] Frenzel C. Teschke R. (2016). Herbal Hepatotoxicity: Clinical Characteristics and Listing Compilation. Int. J. Mol. Sci. 17 (5), 1–38. 10.3390/ijms17050588 PMC4881436 27128912

[B38] Gilani A. H. Janbaz K. H. (1993). Protective effect of Artemisia scoparia extract against acetaminophen-induced hepatotoxicity. Gen. Pharmacol. 24 (6), 1455–1458. 10.1016/0306-3623(93)90434-Y 8112519

[B39] Gilani A. H. Janbaz K. H. (1995a). Preventive and curative effects of Artemisia absinthium on acetaminophen and CCl4-induced hepatotoxicity. Gen. Pharmacol. 26 (2), 309–315. 10.1016/0306-3623(94)00194-R 7590079

[B40] Gilani A. U. Janbaz K. H. (1995b). Studies on protective effect of Cyperus scariosus extract on acetaminophen and CCl4-induced hepatotoxicity. Gen. Pharmacol. 26 (3), 627–631. 10.1016/0306-3623(94)00200-7 7789738

[B41] Goldring C. E. Kitteringham N. R. Elsby R. Randle L. E. Clement Y. N. Williams D. P. (2004). Activation of hepatic Nrf2 in vivo by acetaminophen in CD-1 mice. Hepatol. (Baltimore Md) 39 (5), 1267–1276. 10.1002/hep.20183 15122755

[B42] Gong S. Lan T. Zeng L. Luo H. Yang X. Li N. (2018). Gut microbiota mediates diurnal variation of acetaminophen induced acute liver injury in mice. J. Hepatol. 69 (1), 51–59. 10.1016/j.jhep.2018.02.024 29524531 PMC6365016

[B43] Guimaraes N. S. Mello J. C. Paiva J. S. Bueno P. C. Berretta A. A. Torquato R. J. (2012). Baccharis dracunculifolia, the main source of green propolis, exhibits potent antioxidant activity and prevents oxidative mitochondrial damage. Food Chem. Toxicol. 50 (3-4), 1091–1097. 10.1016/j.fct.2011.11.014 22119782

[B44] Guo Q. Shen Z. Yu H. Lu G. Yu Y. Liu X. (2016). Carnosic acid protects against acetaminophen-induced hepatotoxicity by potentiating Nrf2-mediated antioxidant capacity in mice. Korean J. Physiol. Pharmacol. 20 (1), 15–23. 10.4196/kjpp.2016.20.1.15 26807019 PMC4722187

[B45] Hanafy A. Aldawsari H. M. Badr J. M. Ibrahim A. K. Abdel-Hady Sel S. (2016). Evaluation of Hepatoprotective Activity of Adansonia digitata Extract on Acetaminophen-Induced Hepatotoxicity in Rats. Evid. Based Complement Altern. Med. 2016, 4579149. 10.1155/2016/4579149 PMC4812277 27118980

[B46] Hanawa N. Shinohara M. Saberi B. Gaarde W. A. Han D. Kaplowitz N. (2008). Role of JNK translocation to mitochondria leading to inhibition of mitochondria bioenergetics in acetaminophen-induced liver injury. J. Biol. Chem. 283 (20), 13565–13577. 10.1074/jbc.M708916200 18337250 PMC2376214

[B47] Hau D. K. Gambari R. Wong R. S. Yuen M. C. Cheng G. Y. Tong C. S. (2009). Phyllanthus urinaria extract attenuates acetaminophen induced hepatotoxicity: involvement of cytochrome P450 CYP2E1. Phytomedicine 16 (8), 751–760. 10.1016/j.phymed.2009.01.008 19386480

[B48] Hu J. Ramshesh V. K. McGill M. R. Jaeschke H. Lemasters J. J. (2016). Low Dose Acetaminophen Induces Reversible Mitochondrial Dysfunction Associated with Transient c-Jun N-Terminal Kinase Activation in Mouse Liver. Toxicol. Sci. 150 (1), 204–215. 10.1093/toxsci/kfv319 26721299 PMC5009618

[B49] Huo Y. Yin S. Yan M. Win S. Aung Than T. Aghajan M. (2017). Protective role of p53 in acetaminophen hepatotoxicity. Free Radic. Biol. Med. 106, 111–117. 10.1016/j.freeradbiomed.2017.02.028 28196650 PMC5396540

[B50] Jaeschke H. McGill M. R. Ramachandran A. (2012). Oxidant stress, mitochondria, and cell death mechanisms in drug-induced liver injury: lessons learned from acetaminophen hepatotoxicity. Drug Metab. Rev. 44 (1), 88–106. 10.3109/03602532.2011.602688 22229890 PMC5319847

[B51] Jollow D. J. Mitchell J. R. Potter W. Z. Davis D. C. Gillette J. R. Brodie B. B. (1973). Acetaminophen-induced hepatic necrosis. II. Role of covalent binding in vivo. J. Pharmacol. Exp. Ther. 187 (1), 195–202. 10.1159/000136531 4746327

[B52] Kim S. T. Kim J. D. Ahn S. H. Ahn G. S. Lee Y. I. Jeong Y. S. (2004). Hepatoprotective and antioxidant effects of Alnus japonica extracts on acetaminophen-induced hepatotoxicity in rats. Phytother. Res. 18 (12), 971–975. 10.1002/ptr.1540 15742342

[B53] Kim B. J. Ryu S. W. Song B. J. (2006). JNK- and p38 kinase-mediated phosphorylation of Bax leads to its activation and mitochondrial translocation and to apoptosis of human hepatoma HepG2 cells. J. Biol. Chem. 281 (30), 21256–21265. 10.1074/jbc.M510644200 16709574

[B54] Kim Y. H. Hwang J. H. Kim K. S. Noh J. R. Choi D. H. Kim D. K. (2015). Metformin ameliorates acetaminophen hepatotoxicity via Gadd45beta-dependent regulation of JNK signaling in mice. J. Hepatol. 63 (1), 75–82. 10.1016/j.jhep.2015.02.008 25681557

[B55] Koca-Caliskan U. Yilmaz I. Taslidere A. Yalcin F. N. Aka C. Sekeroglu N. (2018). Cuscuta arvensis Beyr “Dodder”: In Vivo Hepatoprotective Effects Against Acetaminophen-Induced Hepatotoxicity in Rats. J. Med. Food 21 (6), 625–631. 10.1089/jmf.2017.0139 29719159 PMC5998826

[B56] Lakshimarayen V. Muthana M. S. (1953). Essential oil from Premna tomentosa. Indian Inst. Sci. 35, 55–61.

[B57] Lakshmi T. Sri Renukadevi B. Senthilkumar S. Haribalan P. Parameshwari R. Vijayaraghavan R. (2018). Seed and bark extracts of Acacia catechu protects liver from acetaminophen induced hepatotoxicity by modulating oxidative stress, antioxidant enzymes and liver function enzymes in Wistar rat model. BioMed. Pharmacother. 108, 838–844. 10.1016/j.biopha.2018.08.077 30372895

[B58] Lee S. S. Buters J. T. Pineau T. Fernandez-Salguero P. Gonzalez F. J. (1996). Role of CYP2E1 in the hepatotoxicity of acetaminophen. J. Biol. Chem. 271 (20), 12063–12067. 10.1074/jbc.271.20.12063 8662637

[B59] Lee W. M. (2013). Drug-induced acute liver failure. Clin. Liver Dis. 17 (4), 575–586 viii. 10.1016/j.cld.2013.07.001. 24099019 PMC3838908

[B60] Li Y. Z. Ma Z. N. Sun Y. S. Ren S. Jiang S. Zhang W. Z. (2018). Protective effects of extracts of Schisandra chinensis stems against acetaminophen-induced hepatotoxicity via regulation of MAPK and caspase-3 signaling pathways. Chin. J. Natural Medicines 16 (9), 700–713. 10.1016/s1875-5364(18)30110-9 30269847

[B61] Lu Y. Sun J. Petrova K. Yang X. Greenhaw J. Salminen W. F. (2013). Metabolomics evaluation of the effects of green tea extract on acetaminophen-induced hepatotoxicity in mice. Food Chem. Toxicol. 62, 707–721. 10.1016/j.fct.2013.09.025 24080264

[B62] McGill M. R. Jaeschke H. (2013). Metabolism and disposition of acetaminophen: recent advances in relation to hepatotoxicity and diagnosis. Pharm. Res. 30 (9), 2174–2187. 10.1007/s11095-013-1007-6 23462933 PMC3709007

[B63] Mobasher M. A. Gonzalez-Rodriguez A. Santamaria B. Ramos S. Martin M. A. Goya L. (2013). Protein tyrosine phosphatase 1B modulates GSK3beta/Nrf2 and IGFIR signaling pathways in acetaminophen-induced hepatotoxicity. Cell Death Dis. 4, e626. 10.1038/cddis.2013.150 23661004 PMC3674359

[B64] Mroueh M. Saab Y. Rizkallah R. (2004). Hepatoprotective activity of Centaurium erythraea on acetaminophen-induced hepatotoxicity in rats. Phytother. Res. 18 (5), 431–433. 10.1002/ptr.1498 15174008

[B65] Mukazayire M. J. Allaeys V. Buc Calderon P. Stevigny C. Bigendako M. J. Duez P. (2010). Evaluation of the hepatotoxic and hepatoprotective effect of Rwandese herbal drugs on in vivo (guinea pigs barbiturate-induced sleeping time) and in vitro (rat precision-cut liver slices, PCLS) models. Exp. Toxicol. Pathol. 62 (3), 289–299. 10.1016/j.etp.2009.04.005 19493662

[B66] Nakagawa H. Maeda S. Hikiba Y. Ohmae T. Shibata W. Yanai A. (2008). Deletion of apoptosis signal-regulating kinase 1 attenuates acetaminophen-induced liver injury by inhibiting c-Jun N-terminal kinase activation. Gastroenterology 135 (4), 1311–1321. 10.1053/j.gastro.2008.07.006 18700144

[B67] O'Malley G. F. Mizrahi F. Giraldo P. O'Malley R. N. Rollins D. Wilkins D. (2015). Protein-Derived Acetaminophen-Cysteine Can Be Detected After Repeated Supratherapeutic Ingestion of Acetaminophen in the Absence of Hepatotoxicity. J. Med. Toxicol. 11 (3), 317–320. 10.1007/s13181-015-0484-x 26002216 PMC4547951

[B68] Ostapowicz G. Fontana R. J. Schiodt F. V. Larson A. Davern T. J. Han S. H. (2002). Results of a prospective study of acute liver failure at 17 tertiary care centers in the United States. Ann. Int. Med. 137 (12), 947–954. 10.7326/0003-4819-137-12-200212170-00007 12484709

[B69] Pang C. Zheng Z. Shi L. Sheng Y. Wei H. Wang Z. (2016). Caffeic acid prevents acetaminophen-induced liver injury by activating the Keap1-Nrf2 antioxidative defense system. Free Radic. Biol. Med. 91, 236–246. 10.1016/j.freeradbiomed.2015.12.024 26721592

[B70] Parikh H. Pandita N. Khanna A. (2015). Phytoextract of Indian mustard seeds acts by suppressing the generation of ROS against acetaminophen-induced hepatotoxicity in HepG2 cells. Pharm. Biol. 53 (7), 975–984. 10.3109/13880209.2014.950675 25489640

[B71] Patten C. J. Thomas P. E. Guy R. L. Lee M. Gonzalez F. J. Guengerich F. P. (1993). Cytochrome P450 enzymes involved in acetaminophen activation by rat and human liver microsomes and their kinetics. Chem. Res. Toxicol. 6 (4), 511–518. 10.1021/tx00034a019 8374050

[B72] Ramachandran A. Jaeschke H. (2018). Acetaminophen Toxicity: Novel Insights Into Mechanisms and Future Perspectives. Gene Expr. 18 (1), 19–30. 10.3727/105221617x15084371374138 29054140 PMC5885144

[B73] Rezende T. P. do A. C. J. O. Aarestrup B. J. Aarestrup F. M. de Sousa O. V. da Silva Filho A. A. (2014). Protective effects of Baccharis dracunculifolia leaves extract against carbon tetrachloride- and acetaminophen-induced hepatotoxicity in experimental animals. Mol. (Basel Switzerland) 19 (7), 9257–9272. 10.3390/molecules19079257 PMC6271048 24991758

[B74] Roberts D. W. Lee W. M. Hinson J. A. Bai S. Swearingen C. J. Stravitz R. T. (2017). An Immunoassay to Rapidly Measure Acetaminophen Protein Adducts Accurately Identifies Patients With Acute Liver Injury or Failure. Clin. Gastroenterol. Hepatol. 15 (4), 555–62.e3. 10.1016/j.cgh.2016.09.007 27641661 PMC5528860

[B75] Sabate M. Ibanez L. Perez E. Vidal X. Buti M. Xiol X. (2011). Paracetamol in therapeutic dosages and acute liver injury: causality assessment in a prospective case series. BMC Gastroenterol. 11, 80. 10.1186/1471-230x-11-80 21762481 PMC3150324

[B76] Sakeran M. I. Zidan N. Rehman H. Aziz A. T. Saggu S. (2014). Abrogation by Trifolium alexandrinum root extract on hepatotoxicity induced by acetaminophen in rats. Redox Rep. Commun. Free Radical Res. 19 (1), 26–33. 10.1179/1351000213y.0000000068 PMC6837585 24191932

[B77] Salminen W. F. Yang X. Shi Q. Greenhaw J. Davis K. Ali A. A. (2012). Green tea extract can potentiate acetaminophen-induced hepatotoxicity in mice. Food Chem. Toxicol. 50 (5), 1439–1446. 10.1016/j.fct.2012.01.027 22306919

[B78] Sekine S. Lan B. Y. Bedolli M. Feng S. Hebrok M. (2006). Liver-specific loss of beta-catenin blocks glutamine synthesis pathway activity and cytochrome p450 expression in mice. Hepatol. (Baltimore Md) 43 (4), 817–825. 10.1002/hep.21131 16557553

[B79] Shan S. Shen Z. Song F. (2018). Autophagy and acetaminophen-induced hepatotoxicity. Arch. Toxicol. 92 (7), 2153–2161. 10.1007/s00204-018-2237-5 29876591

[B80] Shanmugam S. Thangaraj P. Lima B. D. S. Chandran R. de Souza Araujo A. A. Narain N. (2016). Effects of luteolin and quercetin 3-beta-d-glucoside identified from Passiflora subpeltata leaves against acetaminophen induced hepatotoxicity in rats. BioMed. Pharmacother. 83, 1278–1285. 10.1016/j.biopha.2016.08.044 27567587

[B81] Sharifudin S. A. Fakurazi S. Hidayat M. T. Hairuszah I. Moklas M. A. Arulselvan P. (2013). Therapeutic potential of Moringa oleifera extracts against acetaminophen-induced hepatotoxicity in rats. Pharm. Biol. 51 (3), 279–288. 10.3109/13880209.2012.720993 23043505

[B82] Shon Y. H. Nam K. S. (2004). Protective effect of Moutan Cortex extract on acetaminophen-induced hepatotoxicity in mice. J. Ethnopharmacol. 90 (2-3), 415–419. 10.1016/j.jep.2003.11.004 15013210

[B83] Stickel F. Shouval D. (2015). Hepatotoxicity of herbal and dietary supplements: an update. Arch. Toxicol. 89 (6), 851–865. 10.1007/s00204-015-1471-3 25680499

[B84] Streeter A. J. Dahlin D. C. Nelson S. D. Baillie T. A. (1984). The covalent binding of acetaminophen to protein. Evidence for cysteine residues as major sites of arylation in vitro. Chem. Biol. Interact. 48 (3), 349–366. 10.1016/0009-2797(84)90145-5 6713598

[B85] Tadashi A. Shinji O. Takayaki S. (1990). Triterpenoids, diarylheptanoids and their glycosideses in the flowers of Alnus species. Phytochemistry 29, 3611–3614. 10.1016/0031-9422(90)85286-O

[B86] Teng C. Y. Lai Y. L. Huang H. I. Hsu W. H. Yang C. C. Kuo W. H. (2012). Tournefortia sarmentosa extract attenuates acetaminophen-induced hepatotoxicity. Pharm. Biol. 50 (3), 291–396. 10.3109/13880209.2011.602695 22085220

[B87] Teschke R. Zhu Y. (2018). Paracetamol (Acetaminophen), Alcohol and Liver Injury: Biomarkers, Clinical Issues, and Experimental Aspects. SL Pharmacol. Toxicol. 1 (1), 1–20.

[B88] Thummel K. E. Lee C. A. Kunze K. L. Nelson S. D. Slattery J. T. (1993). Oxidation of acetaminophen to N-acetyl-p-aminobenzoquinone imine by human CYP3A4. Biochem. Pharmacol. 45 (8), 1563–1569. 10.1016/0006-2952(93)90295-8 8387297

[B89] Thummel K. E. Slattery J. T. Ro H. Chien J. Y. Nelson S. D. Lown K. E. (2000). Ethanol and production of the hepatotoxic metabolite of acetaminophen in healthy adults. Clin. Pharmacol. Ther. 67 (6), 591–599. 10.1067/mcp.2000.106574 10872641

[B90] Tien Y. H. Chen B. H. Wang Hsu G. S. Lin W. T. Huang J. H. Lu Y. F. (2014). Hepatoprotective and anti-oxidant activities of Glossogyne tenuifolia against acetaminophen-induced hepatotoxicity in mice. Am. J. Chin. Med. 42 (6), 1385–1398. 10.1142/s0192415x14500876 25384447

[B91] Tong X. Xu J. Lian F. Yu X. Zhao Y. Xu L. (2018). Structural Alteration of Gut Microbiota during the Amelioration of Human Type 2 Diabetes with Hyperlipidemia by Metformin and a Traditional Chinese Herbal Formula: a Multicenter, Randomized, Open Label Clinical Trial. mBio 9 (3), 1–12. 10.1128/mBio.02392-17 PMC5964358 29789365

[B92] Urrunaga N. H. Jadeja R. N. Rachakonda V. Ahmad D. McLean L. P. Cheng K. (2015). M1 muscarinic receptors modify oxidative stress response to acetaminophen-induced acute liver injury. Free Radic. Biol. Med. 78, 66–81. 10.1016/j.freeradbiomed.2014.09.032 25452146 PMC4392405

[B93] Wang Z. Fang J. N. Ge D. L. Li X. Y. (2000). Chemical characterization and immunological activities of an acidic polysaccharide isolated from the seeds of Cuscuta chinensis Lam. Acta Pharmacol. Sin. 21 (12), 1136–1140. 11603289

[B94] Wang X. X. Lv X. Li S. Y. Hou J. Ning J. Wang J. Y. (2015). Identification and characterization of naturally occurring inhibitors against UDP-glucuronosyltransferase 1A1 in Fructus Psoraleae (Bu-gu-zhi). Toxicol. Appl. Pharmacol. 289 (1), 70–78. 10.1016/j.taap.2015.09.003 26348140

[B95] Wang L. Zhang S. Cheng H. Lv H. Cheng G. Ci X. (2016). Nrf2-mediated liver protection by esculentoside A against acetaminophen toxicity through the AMPK/Akt/GSK3beta pathway. Free Radic. Biol. Med. 101, 401–412. 10.1016/j.freeradbiomed.2016.11.009 27836781

[B96] Watkins P. B. Kaplowitz N. Slattery J. T. Colonese C. R. Colucci S. V. Stewart P. W. (2006). Aminotransferase elevations in healthy adults receiving 4 grams of acetaminophen daily: a randomized controlled trial. JAMA 296 (1), 87–93. 10.1001/jama.296.1.87 16820551

[B97] Wei M. Zheng Z. Shi L. Jin Y. Ji L. (2018). Natural Polyphenol Chlorogenic Acid Protects Against Acetaminophen-Induced Hepatotoxicity by Activating ERK/Nrf2 Antioxidative Pathway. Toxicol. Sci. 162 (1), 99–112. 10.1093/toxsci/kfx230 29136249

[B98] Win S. Than T. A. Min R. W. Aghajan M. Kaplowitz N. (2016). c-Jun N-terminal kinase mediates mouse liver injury through a novel Sab (SH3BP5)-dependent pathway leading to inactivation of intramitochondrial Src. Hepatol. (Baltimore Md) 63 (6), 1987–2003. 10.1002/hep.28486 PMC4874901 26845758

[B99] Woodhead J. L. Howell B. A. Yang Y. Harrill A. H. Clewell H. J. 3rd ME A. (2012). An analysis of N-acetylcysteine treatment for acetaminophen overdose using a systems model of drug-induced liver injury. J. Pharmacol. Exp. Ther. 342 (2), 529–540. 10.1124/jpet.112.192930 22593093

[B100] Xie Y. McGill M. R. Cook S. F. Sharpe M. R. Winefield R. D. Wilkins D. G. (2015). Time course of acetaminophen-protein adducts and acetaminophen metabolites in circulation of overdose patients and in HepaRG cells. Xenobiotica 45 (10), 921–929. 10.3109/00498254.2015.1026426 25869248 PMC4553102

[B101] Yamaura K. Shimada M. Nakayama N. Ueno K. (2011). Protective effects of goldenseal (Hydrastis canadensis L.) on acetaminophen-induced hepatotoxicity through inhibition of CYP2E1 in rats. Pharmacogn. Res. 3 (4), 250–255. 10.4103/0974-8490.89745 PMC3249784 22224048

[B102] Yamaura K. Nakayama N. Shimada M. Ueno K. (2012). Protective effects of natsumikan (Citrus natsudaidai) extract on acetaminophen-induced lethal hepatotoxicity in mice. Pharmacogn. Res. 4 (4), 234–236. 10.4103/0974-8490.102274 PMC3510878 23225969

[B103] Ye M. Li Y. Yan Y. Liu H. Ji X. (2002). Determination of flavonoids in Semen Cuscutae by RP-HPLC. J. Pharm. BioMed. Anal. 28 (3-4), 621–628. 10.1016/s0731-7085(01)00672-0 12008141

[B104] Yemitan O. K. Izegbu M. C. (2006). Protective effects of Zingiber officinale (Zingiberaceae) against carbon tetrachloride and acetaminophen-induced hepatotoxicity in rats. Phytother. Res. 20 (11), 997–1002. 10.1002/ptr.1957 16941609

[B105] Yen F. L. Wu T. H. Lin L. T. Lin C. C. (2007). Hepatoprotective and antioxidant effects of Cuscuta chinensis against acetaminophen-induced hepatotoxicity in rats. J. Ethnopharmacol. 111 (1), 123–128. 10.1016/j.jep.2006.11.003 17145147

[B106] Yen F. L. Wu T. H. Lin L. T. Cham T. M. Lin C. C. (2008). Nanoparticles formulation of Cuscuta chinensis prevents acetaminophen-induced hepatotoxicity in rats. Food Chem. Toxicol. 46 (5), 1771–1777. 10.1016/j.fct.2008.01.021 18308443

[B107] Yoon E. Babar A. Choudhary M. Kutner M. Pyrsopoulos N. (2016). Acetaminophen-Induced Hepatotoxicity: a Comprehensive Update. J. Clin. Trans. Hepatol. 4 (2), 131–142. 10.14218/jcth.2015.00052 PMC4913076 27350943

[B108] Yoshioka H. Usuda H. Fujii H. Nonogaki T. (2017). Sasa veitchii extracts suppress acetaminophen-induced hepatotoxicity in mice. Environ. Health Prev. Med. 22 (1), 54. 10.1186/s12199-017-0662-3 29165178 PMC5664914

[B109] Zand R. Nelson S. D. Slattery J. T. Thummel K. E. Kalhorn T. F. Adams S. P. (1993). Inhibition and induction of cytochrome P4502E1-catalyzed oxidation by isoniazid in humans. Clin. Pharmacol. Ther. 54 (2), 142–149. 10.1038/clpt.1993.125 8354023

[B110] Zhang Y. F. He W. Zhang C. Liu X. J. Lu Y. Wang H. (2014). Role of receptor interacting protein (RIP)1 on apoptosis-inducing factor-mediated necroptosis during acetaminophen-evoked acute liver failure in mice. Toxicol. Lett. 225 (3), 445–453. 10.1016/j.toxlet.2014.01.005 24440347

[B111] Zhu J. Chen M. Borlak J. (2019). The landscape of hepatobiliary adverse reactions across 53 herbal and dietary supplements reveals immune-mediated injury as a common cause of hepatitis. Arch. Toxicol. 94 (1), 273–293. 10.1007/s00204-019-02621-4 31720699

